# Special Issue: Structural Concrete Material—New Trends for Eco-Efficiency and Performance

**DOI:** 10.3390/ma15238360

**Published:** 2022-11-24

**Authors:** Eduardo Júlio, Alexandre Bogas, Hugo Costa

**Affiliations:** 1 CERIS, Department of Civil Engineering, Architecture and Georresources (DECivil), Instituto Superior Técnico, Universidade de Lisboa, Av. Rovisco Pais, 1049-001 Lisbon, Portugal; 2 CERIS, Department of Civil Engineering, Coimbra Engineering Institute (Polytechnics of Coimbra), Rua Pedro Nunes, 3030-199 Coimbra, Portugal

During the last few years, structural concrete has experienced significant advances, stimulated by the demand for stricter requirements in terms of sustainability, durability and strength. Extremely relevant developments have been achieved regarding constituents (cement, additions, admixtures and aggregates), mixture design and production techniques, giving rise to a broad set of new structural concretes, e.g., self-compacting, nano-reinforced, fibre-reinforced, lightweight, ultra-high performance and sustainable (low-carbon, low-binder, incorporating recycled aggregates) concretes.

The *fib* Lisbon Symposium 2021 was held in Lisbon from the 14th to the 16th of June 2021, bringing together professionals, researchers and students from all over the world to address ‘Structural Concrete Material—new trends for eco-efficiency and performance’. The main goal was to provide an overview of the latest developments and challenges in the following domains: sustainable cementitious-based materials and alternative binders, including geopolymers, waste cements, alkali-activated cements, modified clinkers and other emerging low-carbon cements; concrete durability and extended lifetime; advanced materials and special concretes; material characterisation; eco-efficiency and life cycle assessment of cementitious-based products.

This Special Issue includes outstanding research from some of the best papers submitted to the *fib* Lisbon Symposium 2021. So far, 11 papers have been published in this Special Issue. A brief summary of each of the presented papers is presented in the following paragraphs.

Pacheco and Brito [1] addressed the use of coarse recycled aggregates, retrieved from heterogeneous construction and demolition waste (CDW), as a partial or total replacement for natural aggregates. Relevant topics, such as CDW composition, methods for the production of recycled aggregates and their influence on the properties of recycled aggregates and recycled aggregate concrete, were discussed. In addition, the paper presents the current requirements for the use of recycled aggregates in concrete.

In another study, within the same scope, Adriana et al. [2] compared the economic and environmental impact of using recycled or natural aggregates in a real-life context, pertaining to the procurement of coarse aggregates for ready-mix concrete plants. Based on case studies, the authors applied the life cycle assessment methodology to different scenarios for the supply of natural and recycled aggregates. The transport distance was found to be a determining factor in the impact of natural and recycled aggregates.

Soldado et al. [3] combined the use of lower binder content and the partial replacement of cement with mineral additions to develop more sustainable low-cement concretes for the production of reinforced concrete poles for electrical distribution lines. These concretes were characterised in terms of their main mechanical and durability properties, and their service life was estimated. When fly ash or pozzolans were adopted as a replacement for cement, low-cement concrete demonstrated a much higher durability than Portland cement concrete.

In their research, Juhart et al. [4] explored the micro-filler/eco-filler concept for achieving a more eco-efficient binder with lower clinker content. Considering particle packing optimisation, a reduction in water demand and optimisation of the mix ratio of the binder blend, a new eco-concrete was derived with satisfactory performance in terms of processability, strength and durability, while showing a reduction of over 20% in carbon emissions compared to standard concrete.

Dybel and Kucharska [5] analysed the viability of multilayer casting in producing low-cement eco-efficient self-compacting concrete (SCC), taking into account different technological scenarios (pouring height, surface treatment of underlying layer, delay time between layers). The general behaviour of low-cement SCC was compared to that of the reference SCC. It was shown that the low-cement SCC was characterised by a lower drop in bond strength between successive layers.

Finally, Antunes et al. [6] experimentally determined the apparent activation energy (E_a_) of a novel low-calcium binder. The E_a_, determined through a calorimetric approach, was correlated with the C/S ratio of the hydration products formed. The effect of temperature on the hydration behaviour and strength development was analysed, and it was found that the novel binder had a higher sensitivity to temperature when compared with Portland cement. 

Tavares and Andrade [7] demonstrated that the usual modelling of chloride penetration, based on Fick’s second law of diffusion, is significantly affected by the presence of reinforcements. In this case, the assumption of semi-infinite boundary conditions is no longer valid, and the chloride concentration at the reinforcement depth becomes higher than that estimated from Fick’s second law. Taking this phenomenon into account, Fick’s second law was numerically solved by a finite element method (FEM). The influence of the reinforcement on the chloride diffusion process depended on the diffusion coefficient, cover depth and steel bar diameter.

Miranda et al. [8] assessed the long-term technical performance (water absorption, carbonation resistance, chloride penetration, shrinkage) and aesthetic compatibility (colour, texture) of restoration mortars for concrete repair. Mortars were produced via the addition of pigments to a white and grey cement-based reference mortar to achieve the required colours. Colour aging was monitored through image processing. It was found a correlation between durability and design parameters. Moreover, service life considering deterioration due to carbonation- and chloride-induced steel corrosion was estimated.

The effect of high temperatures on the bond strength of post-installed rebars (PIRs) made with polymeric mortars was studied by Chehade et al. [9]. The evolution of the pull-out capacity of PIRs was assessed during the post-fire phase after exposure to ISO 834-1 standard fire conditions. The results showed that post-fire pull-out resistance increased with cooling time. A 3D numerical model was considered in calculating the temperature profiles along the embedment depth of the PIR via a transient heat transfer analysis, after exposure to ISO 834 fire followed by cooling to an ambient temperature. The model was then validated with experimental results obtained from a previous experimental study conducted by the authors. Finally, the post-fire pull-out capacity of PIRs in concrete was calculated using Pinoteau’s bond resistance integration method and compared to experimental post-fire pull-out results.

Stindt et al. [10] analysed the effect of rapid heat treatment, which is necessary to accelerate the production speed of precast elements, on the shrinkage and strength of high-performance concrete (HPC) reinforced or not with steel fibres. The results showed that the mechanical strength (compressive, flexural) increased, and the shrinkage significantly reduced with an increasing temperature duration and rebar ratio. The strength loss was compensated for with steel fibres.

Ptacek et al. [11] explored a non-destructive measurement method based on hyperspectral imaging in the near infrared to assess the curing quality of young concrete. The study took into account three different curing types at three different ages. The method showed a high level of reliability for the differentiation between the different curing types and concrete ages.
